# Comparative effectiveness of approved first-line anti-angiogenic and molecularly targeted therapeutic agents in the treatment of good and intermediate risk metastatic clear cell renal cell carcinoma

**DOI:** 10.1186/1471-2407-14-592

**Published:** 2014-08-15

**Authors:** Benjamin Haaland, Akhil Chopra, Sanchalika Acharyya, André P Fay, Gilberto de Lima Lopes

**Affiliations:** Centre for Quantitative Medicine, Office of Clinical Sciences, Duke-National University of Singapore Graduate Medical School, 8 College Road, Singapore, 169857 Singapore; Department of Statistics and Applied Probability, National University of Singapore, Science Drive 2, Singapore, 117546 Singapore; Johns Hopkins Singapore International Medical Center, Jalan Tan Tock Seng, Singapore, 308433 Singapore; Post-Graduate Program - School of Medicine, Pontificia Universidade Catolica do Rio Grande do Sul, Av. Ipiranga, 6681 - Partenon, Porto Alegre, RS 90619-900 Brazil; Dana-Farber Cancer Institute, Lank Center for Genitourinary Oncology, 450 Brookline Ave, Boston, MA 02215-5450 USA; Oncoclinicas do Brasil, Avenida Barbacena 472-14° andar, Belo Horizonte, MG Brazil; Hospital do Coração Cancer Center (HCor Onco), São Paulo, Brazil; Johns Hopkins University, Baltimore, MD USA

**Keywords:** Renal cell carcinoma, VEGF-targeted therapy, Bevacizumab, Sunitinib, Pazopanib, Interferon

## Abstract

**Background:**

Based on improved clinical outcomes in randomized controlled clinical trials (RCTs) the FDA and EMA have approved bevacizumab with interferon, sunitinib, and pazopanib in the first-line treatment of low to intermediate risk metastatic clear cell renal cell carcinoma (mRCC). However, there is little comparative data to help in choosing the most effective drug among these agents.

**Methods:**

We performed an indirect comparative effectiveness analysis of the pivotal RCTs of bevacizumab with interferon, sunitinib, or pazopanib compared to one another or interferon alone in first-line treatment of metastatic or advanced RCC. Endpoints of interest were overall survival (OS), progression free survival (PFS), and response rate (RR). Adverse events were also examined.

**Results:**

The meta-estimate of the hazard ratio (95% confidence interval) for OS for bevacizumab with interferon vs. interferon alone was 0.86 (0.76-0.97), for sunitinib vs. interferon alone was 0.82 (0.67-1.00), for pazopanib vs. interferon alone was 0.74 (0.57-0.97), for sunitinib vs. bevacizumab with interferon was 0.95 (0.75-1.20), for pazopanib vs. bevacizumab with interferon was 0.86 (0.64-1.16), and for pazopanib vs. sunitinib was 0.91 (0.76-1.08). Similarly, bevacizumab with interferon, sunitinib, or pazopanib had better PFS and RR than interferon alone. Sunitinib and pazopanib had better RR than bevacizumab with interferon and there was suggestive evidence pazopanib may outperform sunitinib in terms of RR.

**Conclusions:**

Bevacizumab with interferon, sunitinib, and pazopanib are adequate first-line options in treatment of mRCC. Interferon alone should not be considered an optimal first-line treatment.

**Electronic supplementary material:**

The online version of this article (doi:10.1186/1471-2407-14-592) contains supplementary material, which is available to authorized users.

## Background

Approximately 64,000 new cases of kidney cancer are diagnosed each year in the United States and 25%-30% of these result in death [1]. RCC accounts for 80-90% of kidney cancers and 70-80% of these are clear cell RCC [2]. Surgery is curative in the majority of patients with local disease. However, local recurrence or distant metastasis occur in up to 40% of patients treated for localized tumors and 5-year survival is less than 10% in this subgroup [2–4].

RCC is characterized by a high degree of resistance to chemotherapy. Historically, tumors have been treated with cytokines with modest RR and small survival benefit [5]. High-dose interleukin-2 remains an option for highly selected patients and is associated with durable remission in a small minority of patients [6, 7].

The biology underlying RCC has been elucidated [8]. Mutations in the Von Hippel-Lindau (*VHL)* gene are present in most cases of sporadic RCC [9]. When *VHL* is inactivated, there is an up-regulation of hypoxia-inducible factors (HIFs) and subsequent activation of pathways involved with metabolism, inflammation, and angiogenesis [9–11]. This rationale has provided a theoretical basis for the development of several agents targeting angiogenesis, including vascular endothelial growth factor (VEGF) and mammalian target of rapamycin (mTOR) [12].

Since 2005 the US Food and Drug Administration (FDA) and European Medicines Agency (EMA) have approved novel agents targeting the VEGF-pathway for patients with mRCC based on large and well-powered randomized clinical trials. Motzer et al. reported that sunitinib (an oral VEGF tyrosine kinase inhibitors) improves PFS compared with interferon-alfa [13, 14]. Two studies evaluated the role of bevacizumab (an intravenous antibody against VEGF) in first-line treatment of mRCC: Rini et al. reported an improvement in PFS and a trend towards better OS in patients treated with bevacizumab plus interferon alfa compared with interferon alfa alone [15, 16] while Escudier et al. (AVOREN trial) corroborated the results for PFS in the arm treated with both drugs [17, 18]. In addition, Motzer et al. showed non-inferiority of pazopanib (another oral VEGF tyrosine kinase inhibitors) to sunitinib in terms of PFS [19].

Although several agents were successfully developed and have become the standard of care in treatment of advanced RCC, the selection of appropriate treatment is based on clinical setting (previously treated or previously untreated patients), prognostic stratification (good/intermediate or poor), and histology [8]. However, there is little if any comparative data to help choose the most effective drug to improve patients’ outcomes, and predictive biomarkers of treatment response are also lacking [20].

We sought to conduct a meta-comparison of pivotal RCTs in the first-line treatment of metastatic clear cell RCC in order to establish the most effective therapy in this setting.

## Methods

We performed a meta-comparison of the 4 pivotal RCTs to evaluate the effectiveness of first-line agents in the treatment of mRCC in patients with good to intermediate risk.

### Evidence acquisition

A systematic literature search was performed targeting publications reporting on randomized phase 3 clinical trials comparing bevacizumab with interferon, sunitinib, or pazopanib to one another or interferon alone as first-line therapy for patients with good to intermediate risk metastatic or advanced renal clear cell carcinoma. Medline was searched through PubMed using the search phrase (“sunitinib” OR “bevacizumab” OR “pazopanib”) AND (“renal cell carcinoma” OR “renal-cell carcinoma”) AND (“advanced” OR “metastatic”) limited to clinical trials during the last 10 years. Supplemental searches of the 2014 and 2013 ASCO Annual Meetings and Genitourinary Cancers Symposiums [21] as well as clinicaltrials.gov [22] were also performed. Two reviewers independently screened the titles and abstracts of the identified studies and the full texts of all potentially relevant studies. Comparative estimates from the studies that fulfilled all inclusion criteria were extracted in a standardized form with disagreements resolved by consensus.

### Statistical analysis

Meta-analysis for efficacy outcomes was performed in the context of linear mixed effects models, with random effects for each study and fixed effects for each study’s specific treatment contrast, based on comparative estimates extracted from each study. Estimates, confidence intervals, and p-values from analyses stratified by risk factors were used throughout if available. The linear mixed effects model for meta-analysis is a generalization of the meta-analysis models proposed in DerSimonian et al. [23] within which meta-regression techniques [24, 25] can be used to compare treatments and estimate study-to-study heterogeneity. In particular, let  denote the vector of treatment contrast estimates (log hazard or odds ratios), let *X* denote the design matrix with each row containing the treatment contrast associated with the particular component of *y*, and let  denote the diagonal matrix with the treatment contrast variance estimates. An *I*^*2*^ statistic measuring heterogeneity in treatment contrasts across studies and having an interpretation similar to intra-class correlation was developed in a manner similar to Higgins et al. [26]. In particular, a goodness-of-fit statistic is calculated as *Q* = *y* ' *W*^− 1^(*I* − *H*)*y*, where *I* denotes a *K* dimensional identity matrix and *H* = *X*(*X* ' *W*^− 1^*X*)^− 1^*X* ' *W*^− 1^ denotes a weighted projection into the column space of the design matrix *X*. Under the hypothesis that there is no study-to-study heterogeneity H_0_ : *σ*^2^ = 0, *Q* has a chi-squared distribution , where rank(*X*) denotes the number of linearly independent columns in *X*. The *I*^2^ measure of heterogeneity is then the greater of (*Q* − (*K* − rank(*X*)))/*Q* and zero. The study-to-study variability can be estimated by equating the sample value of *Q* to its expectation and truncating at zero, giving


where trace {*A*} denotes the sum of the diagonal elements of *A*. Then, each *estimable* meta-estimate is given by , where  and , with variance estimate . Tests of heterogeneity and *I*^2^ can be misleading when treatments differ markedly even in the presence of study-to-study heterogeneity. Predictive intervals provide an interval in which a specific site’s relative efficacy can be expected to fall and were computed using the study-to-study variance estimates. Pooling of adverse event rates was performed separately for each treatment under the assumption of no study-to-study heterogeneity. All statistical analyses were performed in R 3.0.1 (R Development Core Team, 2012).

## Results

### Search results

**Figure 1:**
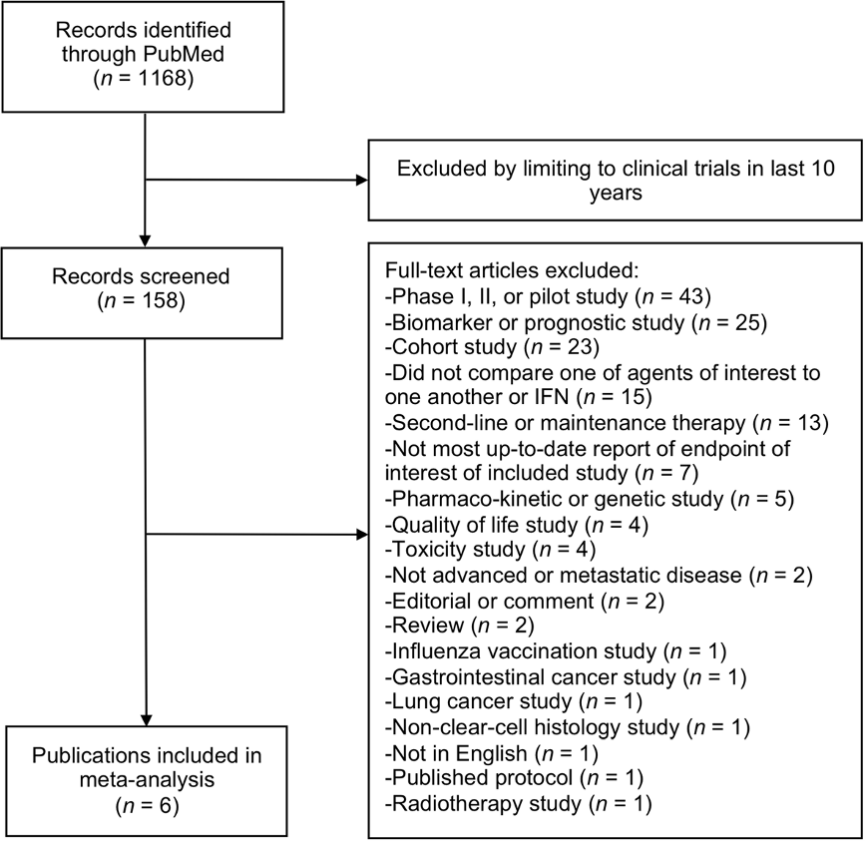
**Selection diagram for studies comparing bevacizumab with interferon, sunitinib, and pazopanib to interferon alone or one another as first-line therapy for patients with clear cell renal cell carcinoma.**

The search identified 6 publications on 4 studies comparing bevacizumab with interferon, sunitinib, or pazopanib to one another or interferon alone as first-line treatment in patients with metastatic or advanced clear cell renal cell carcinoma. The search is summarized in Figure 1.

The identified studies were Motzer et al. [14] comparing sunitinib to interferon alone, Rini et al. (CALGB 90206) [15, 16] and Escudier et al. (AVOREN) [17, 18] comparing bevacizumab with interferon to interferon alone, and Motzer et al. (COMPARZ) [19] comparing pazopanib to sunitinib. The most up-to-date reports on overall survival in the CALGB 90206 and AVOREN trials were in Rini et al. [16] and Escudier et al. [18]. All studies included adult patients with good or intermediate risk advanced or metastatic renal cell carcinoma with a clear cell histological component that had not received prior systemic therapy. Treatment arms, sample size, and results for included studies are summarized in Table 1.

**Table 1:** **Summary of included trials comparing bevacizumab with interferon (Bev + IFN), sunitinib, and pazopanib to interferon alone (IFN) or one another as first-line therapy for patients with clear cell renal cell carcinoma**

Trial	Treatment arms (n)	Overall survival		Progression-free survival		Response	
		Median ^a^	HR (95% CI)	Median ^a^	HR (95% CI)	Percent	OR (95% CI)
Rini et al. (2008; 2013) [15, 16]	Bev + IFN^b,c^ (n = 369)	18.3	0.86 (0.73-1.01)	8.5	0.71 (0.61-0.83)	26%	2.27 (1.51-3.42)
	IFN^c^ (n = 363)	17.4		5.2		13%	
Escudier et al. (2007; 2010) [17, 18]	Bev + IFN^b,c^ (n = 327)	23.3	0.86 (0.72-1.04)	10.2	0.61 (0.51-0.73)	31%^f^	3.11 (2.04-4.74)
	IFN^c^ (n = 322)	21.3		5.4		13%^f^	
Motzer et al. (2013) [19]	Pazopanib^d^ (n = 557)	28.4	0.91 (0.76-1.08)	8.4	1.05 (0.90-1.22)	31%	1.35 (1.03-1.75)
	Sunitinib^e^ (n = 553)	29.3		9.5		25%	
Motzer et al. (2007; 2009) [14]	Sunitinib^e^ (n = 375)	26.4	0.82 (0.67-1.00)	11	0.54 (0.45-0.64)	47%	6.33 (4.37-9.15)
	IFN^c^ (n = 375)	21.8	5		12%		

^a^months.

^b^bevucizumab 10 mg/kg every 2 weeks.

^c^interferon alfa 9 million units subcutaneously three times weekly.

^d^pazopanib 800 mg once daily.

^e^sunitinib 50 mg once daily for 4 weeks, followed by 2 weeks off.

^f^denominator for Bev + IFN 306, denominator for IFN + Placebo 289.

### Overall survival

**Figure 2:**
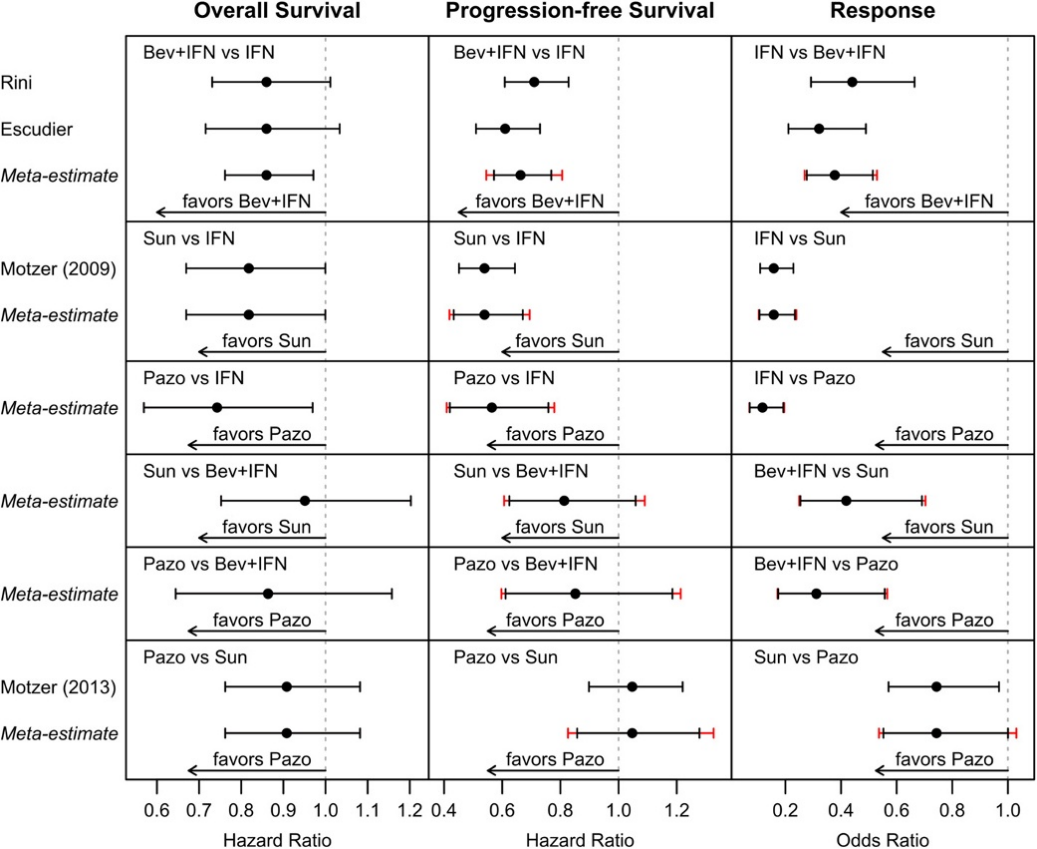
**Individual study and comparative meta-estimate hazard ratios and odds ratios for overall survival, progression-free survival, and response for bevacizumab with interferon (Bev + IFN), sunitinib (Sun), pazopanib (Pazo), and interferon alone (IFN) as first-line therapy for patients with clear cell renal cell carcinoma.**

The test of heterogeneity indicated low study-to-study variability with *Q* = 0 on 1 degree of freedom (*p* = 1) and *I*^2^ = 0%. The overall survival hazard ratio meta-estimate (95% confidence interval; 95% prediction interval) for bevacizumab with interferon vs. interferon alone was 0.86 (0.76-0.97; 0.76-0.97), for sunitinib vs. interferon alone was 0.82 (0.67-1.00; 0.67-1.00), for pazopanib vs. interferon alone was 0.74 (0.57-0.97; 0.57-0.97), for sunitinib vs. bevacizumab with interferon was 0.95 (0.75-1.20; 0.75-1.20), for pazopanib vs. bevacizumab with interferon was 0.86 (0.64-1.16; 0.64-1.16), and for pazopanib vs. sunitinib was 0.91 (0.76-1.08; 0.76-1.08). These results are summarized in Table 2 and Figure 2.

**Table 2:** **Meta-comparisons of bevacizumab with interferon (Bev + IFN), sunitinib (Sun), pazopanib (Pazo), and interferon alone (IFN) as first-line therapy for patients with clear cell renal cell carcinoma**

Comparison	Overall survival	Progression-free survival	Response
	HR (95% CI; 95% PI)	HR (95% CI; 95% PI)	OR (95% CI; 95% PI)
Bev + IFN vs IFN	0.86 (0.76-0.97; 0.76-0.97)	0.66 (0.57-0.77; 0.55-0.81)	2.65 (1.94-3.61; 1.89-3.71)
Sun vs IFN	0.82 (0.67-1.00; 0.67-1.00)	0.54 (0.43-0.67; 0.42-0.69)	6.33 (4.27-9.37; 4.17-9.59)
Pazo vs IFN	0.74 (0.57-0.97; 0.57-0.97)	0.56 (0.42-0.76; 0.41-0.78)	8.51 (5.20-13.93; 5.10-14.19)
Sun vs Bev + IFN	0.95 (0.75-1.20; 0.75-1.20)	0.81 (0.62-1.06; 0.61-1.09)	2.39 (1.45-3.94; 1.42-4.01)
Pazo vs Bev + IFN	0.86 (0.64-1.16; 0.64-1.16)	0.85 (0.61-1.19; 0.60-1.21)	3.21 (1.79-5.75; 1.77-5.84)
Pazo vs Sun	0.91 (0.76-1.08; 0.76-1.08)	1.05 (0.86-1.28; 0.83-1.33)	1.35 (1.00-1.81; 0.97-1.86)

### Progression free survival

The test of heterogeneity indicated moderate study-to-study variability with *Q* = 1.58 on 1 degree of freedom (*p* = 0.208) and *I*^2^ = 37%. The progression-free survival hazard ratio meta-estimate (95% confidence interval; 95% prediction interval) for bevacizumab with interferon vs. interferon alone was 0.66 (0.57-0.77; 0.55-0.81), for sunitinib vs. interferon alone was 0.54 (0.43-0.67; 0.42-0.69), for pazopanib vs. interferon alone was 0.56 (0.42-0.76; 0.41-0.78), for sunitinib vs. bevacizumab with interferon was 0.81 (0.62-1.06; 0.61-1.09), for pazopanib vs. bevacizumab with interferon was 0.85 (0.61-1.19; 0.60-1.21), and for pazopanib vs. sunitinib was 1.05 (0.86-1.28; 0.83-1.33). These results are summarized in Table 2 and Figure 2.

### Response rate

The test of heterogeneity indicated low study-to-study variability with *Q* = 1.11 on 1 degree of freedom (*p* = 0.293) and *I*^2^ = 10%. The response rate odds ratio meta-estimate (95% confidence interval; 95% prediction interval) for bevacizumab with interferon vs. interferon alone was 2.65 (1.94-3.61; 1.89-3.71), for sunitinib vs. interferon alone was 6.33 (4.27-9.37; 4.17-9.59), for pazopanib vs. interferon alone was 8.51 (5.20-13.93; 5.10-14.19), for sunitinib vs. bevacizumab with interferon was 2.39 (1.45-3.94; 1.42-4.01), for pazopanib vs. bevacizumab with interferon was 3.21 (1.79-5.75; 1.77-5.84), and for pazopanib vs. sunitinib was 1.35 (1.00-1.81; 0.97-1.86). These results are summarized in Table 2 and Figure 2.

### Adverse events

Broadly, adverse event rates were lower for interferon than for bevacizumab with interferon, sunitinib, or pazopanib, while adverse event rates were similar for bevacizumab with interferon, sunitinib, and pazopanib. In particular, grade 3 or worse adverse events rates (95% confidence intervals) for interferon alone, bevacizumab with interferon, sunitinib, and pazopanib were 0.544 (0.505-0.582), 0.705 (0.670-0.738), 0.734 (0.695-0.769), and 0.744 (0.706-0.778), respectively. Adverse event rates are summarized in brief in Table 3 and completely for all reported adverse events in Additional file 1: Table S1.

**Table 3:** **Adverse event rates by approved first-line anti-angiogenic and molecularly targeted therapeutic agents in the treatment of good and intermediate risk metastatic clear cell renal cell carcinoma**

	Interferon	Bevacizumab with interferon	Sunitinib	Pazopanib
	Rate (95% CI)	Rate (95% CI)	Rate (95% CI)	Rate (95% CI)
Any grade 3, 4, or 5	0.544 (0.505-0.582)	0.705 (0.670-0.738)	0.734 (0.695-0.769)	0.744 (0.706-0.778)
AE leading to discontinuation of drug	0.185 (0.158-0.217)	0.282 (0.237-0.332)	0.197 (0.173-0.224)	0.244 (0.210-0.281)
AE leading to death	0.013 (0.008-0.022)	0.016 (0.009-0.028)	0.022 (0.014-0.033)	0.023 (0.014-0.040)
Thrombocytopenia	0.135 (0.115-0.157)	0.084 (0.066-0.107)	0.732 (0.703-0.760)	0.410 (0.370-0.451)
Grade ≥ 3	0.009 (0.005-0.017)	0.021 (0.013-0.035)	0.164 (0.141-0.189)	0.036 (0.023-0.055)
Neutropenia	0.320 (0.292-0.350)	0.260 (0.229-0.294)	0.714 (0.684-0.742)	0.366 (0.327-0.407)
Grade ≥ 3	0.069 (0.055-0.087)	0.069 (0.052-0.090)	0.192 (0.168-0.218)	0.045 (0.031-0.066)
Anemia	0.365 (0.336-0.395)	0.132 (0.109-0.159)	0.674 (0.643-0.703)	0.309 (0.272-0.348)
Grade ≥ 3	0.051 (0.039-0.067)	0.033 (0.022-0.049)	0.076 (0.060-0.095)	0.022 (0.012-0.037)
Asthenic conditions or fatigue	0.576 (0.545-0.606)	0.638 (0.602-0.673)	0.593 (0.561-0.624)	0.545 (0.503-0.586)
Grade ≥ 3	0.178 (0.156-0.203)	0.250 (0.220-0.284)	0.146 (0.125-0.171)	0.106 (0.083-0.135)
Diarrhea	0.152 (0.127-0.181)	0.205 (0.165-0.251)	0.589 (0.557-0.621)	0.628 (0.587-0.667)
Grade ≥ 3	0.011 (0.005-0.022)	0.021 (0.010-0.042)	0.082 (0.066-0.102)	0.088 (0.068-0.115)
Nausea	0.467 (0.430-0.504)	0.580 (0.529-0.630)	0.482 (0.450-0.514)	0.446 (0.405-0.487)
Grade ≥ 3	0.030 (0.020-0.045)	0.072 (0.049-0.103)	0.034 (0.024-0.047)	0.022 (0.012-0.037)
Anorexia or appetite loss	0.402 (0.372-0.432)	0.542 (0.505-0.579)	0.358 (0.327-0.389)	0.374 (0.334-0.415)
Grade ≥ 3	0.043 (0.032-0.057)	0.104 (0.084-0.129)	0.029 (0.020-0.042)	0.014 (0.007-0.028)
HTN	0.054 (0.042-0.070)	0.273 (0.242-0.307)	0.364 (0.334-0.396)	0.464 (0.423-0.506)
Grade ≥ 3	0.006 (0.003-0.013)	0.072 (0.055-0.093)	0.137 (0.116-0.160)	0.148 (0.121-0.180)
Proteinuria	0.049 (0.035-0.069)	0.452 (0.416-0.489)	0.137 (0.111-0.168)	0.177 (0.147-0.211)
Grade ≥ 3	0.002 (0.000-0.009)	0.112 (0.090-0.137)	0.040 (0.027-0.060)	0.042 (0.028-0.062)
Pyrexia	0.386 (0.349-0.423)	0.451 (0.399-0.504)	0.128 (0.108-0.151)	0.087 (0.066-0.113)
Grade ≥ 3	0.009 (0.004-0.020)	0.024 (0.012-0.046)	0.011 (0.006-0.020)	0.004 (0.001-0.013)
Headache	0.161 (0.135-0.191)	0.234 (0.192-0.282)	0.186 (0.163-0.213)	0.227 (0.194-0.264)
Grade ≥ 3	0.006 (0.002-0.015)	0.021 (0.010-0.042)	0.011 (0.006-0.020)	0.027 (0.016-0.044)
Thyroid dysfunction	0.010 (0.005-0.020)	0.006 (0.002-0.020)	0.202 (0.177-0.229)	0.121 (0.096-0.151)
Grade ≥ 3	0.006 (0.002-0.014)	0.006 (0.002-0.020)	0.011 (0.006-0.020)	0.000 (0.000-0.007)
Weight loss	0.130 (0.107-0.157)	0.157 (0.124-0.199)	0.085 (0.068-0.104)	0.152 (0.124-0.184)
Grade ≥ 3	0.013 (0.007-0.024)	0.041 (0.025-0.067)	0.005 (0.002-0.013)	0.009 (0.004-0.021)
Dyspnea	0.098 (0.081-0.118)	0.139 (0.115-0.166)	0.143 (0.122-0.167)	0.137 (0.111-0.168)
Grade ≥ 3	0.026 (0.018-0.037)	0.036 (0.024-0.052)	0.023 (0.015-0.035)	0.025 (0.015-0.042)

## Discussion

The treatment of mRCC has evolved over the last 9 years and the list of first-line targeted therapies is ever increasing [20]. Sunitinib, pazopanib, and bevacizumab plus interferon have demonstrated convincing clinical benefit in patients with favorable or intermediate prognosis [13–19, 27]. These new interventions have been evaluated, compared to interferon or one another as first-line treatment but there are limited phase 3 trials providing data comparing different treatments.

At present, the selection of appropriate treatment is based on prognostic risk category, available PFS and OS data, and toxicity profile. The most widely used prognostic tool is the Memorial Sloan Kettering Cancer Center (MSKCC) model, which stratifies prognosis as good, intermediate or poor, based on high lactate dehydrogenase, low Karnofsky score, high corrected calcium, low hemoglobin and shorter time from diagnosis to treatment [28, 29]. In the era of targeted therapy, the International mRCC Database Consortium (IMDC) prognostic model has been used to stratify patients according to the presence of six adverse prognostic factors: Karnofsky score <80%, low hemoglobin, time from diagnosis to treatment of <1 year, high corrected calcium, thrombocytosis, and neutrophilia [8, 20]. In addition, histology (clear cell vs. non-clear cell), personal experience and cost are also important considerations in the decision-making process [30].

The intent of our study was to perform meta-comparison of the pivotal RCTs to provide evidence on the best first-line treatment of patients with good to intermediate risk mRCC. OS, PFS, and RR favored the use of bevacizumab with interferon, sunitinib, or pazopanib when compared to interferon alone. There was evidence that sunitinib and pazopanib outperformed bevacizumab with interferon in terms of RR, while there was suggestive evidence that RR may be better with pazopanib than sunitinib. While there was a low to moderate heterogeneity across studies in efficacy endpoints, comparative results should be interpreted cautiously.

A number of related studies did not meet inclusion criteria, but were also of interest. Sternberg et al. demonstrated the efficacy of pazopanib as compared to placebo in PFS improvement [31, 32]. Hutson et al. failed to show a statistically significant PFS benefit for axitinib over sorefenib as first-line treatment in patients with mRCC [33]. Randomized trials showed superiority of sorafenib over placebo in second-line therapy in a phase 3 trial, but not over interferon as first-line therapy in a phase 2 trial [34, 35]. Several older studies, MRCRCC [36], Kriegmair et al. [37], Pyrhonen et al. [38], and Steineck et al. [39], provided evidence largely favoring interferon over controls. These studies were not incorporated in the present meta-comparison in spite of their importance, due to the potential for bias in the present day context as continual advances in supportive care may have altered the relative effectiveness of treatments.

Initially, phase 2 studies and studies comparing one of the treatments of interest to a control were considered for the present comparison. The broader search identified two additional studies, the phase 2 TORAVA study comparing temsirolimus and bevacizumab, sunitinib, and bevacizumab with interferon [40] and the phase 3 Sternberg et al. study comparing pazopanib to placebo [31, 32]. However, the TORAVA study did not report hazard ratios for overall or progression-free survival and contained 12% poor risk patients. The Sternberg et al. study, on the other hand, did not add information on the comparative effectiveness of bevacizumab with interferon, sunitinib, and pazopanib, as it was the only relatively recent study that compared an agent of interest to control.

A meta-analysis of seven RCTs that evaluated sunitinib, bevacizumab with interferon, or sorafenib compared with interferon or placebo showed that anti-VEGF agents significantly prolonged PFS and offered important clinical benefits to patients with mRCC. Among these drugs, sunitinib had higher RR [41]. Interestingly, Mills and colleagues reported an indirect comparison from 5 full-length articles and 2 abstracts that evaluate these same drugs. Using interferon as the control arm, they showed that sunitinib was superior to both sorafenib (HR 0.58, 95% CI, 0.38–0.86, p < 0.001) and bevacizumab with interferon (HR 0.75, 95% CI, 0.60–0.93, p = 0.001). Sorafenib was not statistically different from bevacizumab with interferon [42]. However, both of these studies included phase 2 and second-line studies, as well as studies on drugs not commonly used in patients with good to intermediate risk.

The PISCES study compared patient preference for pazopanib and sunitinib as first-line treatment of mRCC in the context of a randomized crossover trial, and found that 70% of patients preferred treatment with pazopanib because of reductions in fatigue and improved quality of life [43]. In addition, Cella and colleagues reported quality-of-life in favor of pazopanib over sunitinib in the COMPARZ study [44]. We found that adverse event rates were lower for interferon than for bevacizumab with interferon, sunitinib, or pazopanib, while adverse event rates were similar for bevacizumab with interferon, sunitinib, and pazopanib.

Tolerability is an important consideration in selecting therapy for mRCC with increasing patient survival and long-term use of therapy [45]. A recent meta-analysis demonstrated that bevacizumab is asscociated with an increase of 33% in fatal adverse events compared with chemotherapy alone [46]. Furthermore, Schutz et al. reported that use of VEGF tyrosine kinase inhibitors was associated with increased risk of fatal adverse events [47]. Patient comorbidities are also important considerations in treatment selection.

Novel agents for advanced RCC require selection paradigms to optimize first-line therapy. Recently, Choueiri and colleagues evaluated several potential biomarkers along the VHL/HIF1α/HIF2α axis and none of them were found predictive of pazopanib activity [48]. Currently, there are no clinical factors or biomarkers that can reliably predict which targeted therapies patients will respond to.

Our study has limitations. Direct comparisons remain the highest level of evidence of therapeutic effectiveness and our results must be interpreted with caution since several are based on indirect comparison. Further, despite the fact that all selected RCTs were of high quality, agents were evaluated in slightly different clinical settings and populations. In addition, all other factors equals, individual patient data (IPD) meta-analyses are preferable to aggregated data meta-analyses because IPD allows for subgroup analyses, inclusion of inappropriately excluded patients, data checking, randomization checking, verification of analyses, and potentially more long-term and uniform follow-up [49]. However, in the current context, the main results are not likely to be altered meaningfully by using IPD, as all included efficacy data is intention-to-treat, based on simple and standard analyses, and all included studies are relatively high-quality in terms of trial execution and outcomes assessment.

## Conclusions

In summary, several studies support VEGF-targeted therapies as the standard of mRCC treatment. Our analysis provides a comparison on the basis of the pivotal RCTs and demonstrates that any of bevacizumab with interferon, sunitinib, and pazopanib offer improved survival and substantial clinical benefits in comparison with interferon alone. Efforts to identify predictive biomarkers for treatment response and direct comparisons among the drugs are needed to customize therapy in mRCC.

## Electronic supplementary material

Additional file 1: Table S1: Adverse event rates by approved front-line anti-angiogenic and molecularly targeted therapeutic agents in the treatment of good and intermediate risk metastatic clear cell renal cell carcinoma. (DOCX 27 KB)
