# Supplementary material for: Comparative effectiveness of approved first-line anti-angiogenic and molecularly targeted therapeutic agents in the treatment of good and intermediate risk metastatic clear cell renal cell carcinoma
Source: BMC Cancer. 2014 Aug 15;14:592. doi: 10.1186/1471-2407-14-592 (PMC4148555; doi:10.1186/1471-2407-14-592)
Supplement: Supplementary file 1 — Additional file 1: Table S1: Adverse event rates by approved front-line anti-angiogenic and molecularly targeted therapeutic agents in the treatment of good and intermediate risk metastatic clear cell renal cell carcinoma. (DOCX 27 KB) [file 12885_2014_4780_MOESM1_ESM.docx]

Additional file 1: Table S1: Adverse event rates by approved front-line anti-angiogenic and molecularly targeted therapeutic agents in the treatment of good and intermediate risk metastatic clear cell renal cell carcinoma

| Adverse event | Interferon | Bevacizumab with interferon | Sunitinib | Pazopanib |
| --- | --- | --- | --- | --- |
|  | Rate (95% CI) | Rate (95% CI) | Rate (95% CI) | Rate (95% CI) |
| Leukopenia | 0.569 (0.518-0.620) | - | 0.776 (0.748-0.801) | 0.428 (0.387-0.469) |
| Grade>=3 | 0.019 (0.009-0.040) | - | 0.069 (0.055-0.088) | 0.014 (0.007-0.028) |
| Thrombocytopenia | 0.135 (0.115-0.157) | 0.084 (0.066-0.107) | 0.732 (0.703-0.760) | 0.410 (0.370-0.451) |
| Grade>=3 | 0.009 (0.005-0.017) | 0.021 (0.013-0.035) | 0.164 (0.141-0.189) | 0.036 (0.023-0.055) |
| Neutropenia | 0.320 (0.292-0.350) | 0.260 (0.229-0.294) | 0.714 (0.684-0.742) | 0.366 (0.327-0.407) |
| Grade>=3 | 0.069 (0.055-0.087) | 0.069 (0.052-0.090) | 0.192 (0.168-0.218) | 0.045 (0.031-0.066) |
| Lymphopenia | 0.689 (0.639-0.735) | - | 0.601 (0.569-0.632) | 0.375 (0.336-0.417) |
| Grade>=3 | 0.261 (0.218-0.309) | - | 0.157 (0.135-0.182) | 0.052 (0.037-0.074) |
| Anemia | 0.365 (0.336-0.395) | 0.132 (0.109-0.159) | 0.674 (0.643-0.703) | 0.309 (0.272-0.348) |
| Grade>=3 | 0.051 (0.039-0.067) | 0.033 (0.022-0.049) | 0.076 (0.060-0.095) | 0.022 (0.012-0.037) |
| Asthenic conditions or fatigue | 0.576 (0.545-0.606) | 0.638 (0.602-0.673) | 0.593 (0.561-0.624) | 0.545 (0.503-0.586) |
| Grade>=3 | 0.178 (0.156-0.203) | 0.250 (0.220-0.284) | 0.146 (0.125-0.171) | 0.106 (0.083-0.135) |
| Diarrhea | 0.152 (0.127-0.181) | 0.205 (0.165-0.251) | 0.589 (0.557-0.621) | 0.628 (0.587-0.667) |
| Grade>=3 | 0.011 (0.005-0.022) | 0.021 (0.010-0.042) | 0.082 (0.066-0.102) | 0.088 (0.068-0.115) |
| AST elevation | 0.381 (0.332-0.432) | - | 0.577 (0.545-0.609) | 0.601 (0.560-0.641) |
| Grade>=3 | 0.019 (0.009-0.040) | - | 0.025 (0.017-0.037) | 0.125 (0.100-0.155) |
| ALT elevation | 0.400 (0.351-0.451) | - | 0.460 (0.429-0.493) | 0.588 (0.547-0.629) |
| Grade>=3 | 0.019 (0.009-0.040) | - | 0.035 (0.025-0.049) | 0.173 (0.144-0.207) |
| Nausea | 0.467 (0.430-0.504) | 0.580 (0.529-0.630) | 0.482 (0.450-0.514) | 0.446 (0.405-0.487) |
| Grade>=3 | 0.030 (0.020-0.045) | 0.072 (0.049-0.103) | 0.034 (0.024-0.047) | 0.022 (0.012-0.037) |
| Hyperglycemia | - | - | 0.560 (0.518-0.601) | 0.531 (0.489-0.572) |
| Grade>=3 | - | - | 0.040 (0.027-0.060) | 0.051 (0.035-0.072) |
| Increased lipase | 0.461 (0.410-0.513) | - | 0.560 (0.509-0.609) | - |
| Grade>=3 | 0.081 (0.057-0.113) | - | 0.181 (0.146-0.223) | - |
| Creatinine elevation | 0.511 (0.460-0.562) | - | 0.556 (0.524-0.588) | 0.319 (0.282-0.359) |
| Grade>=3 | 0.011 (0.004-0.028) | - | 0.017 (0.011-0.028) | 0.007 (0.003-0.018) |
| Anorexia or appetite loss | 0.402 (0.372-0.432) | 0.542 (0.505-0.579) | 0.358 (0.327-0.389) | 0.374 (0.334-0.415) |
| Grade>=3 | 0.043 (0.032-0.057) | 0.104 (0.084-0.129) | 0.029 (0.020-0.042) | 0.014 (0.007-0.028) |
| Increased creatine kinase | 0.119 (0.090-0.157) | - | 0.491 (0.440-0.541) | - |
| Grade>=3 | 0.011 (0.004-0.028) | - | 0.029 (0.016-0.052) | - |
| HTN | 0.054 (0.042-0.070) | 0.273 (0.242-0.307) | 0.364 (0.334-0.396) | 0.464 (0.423-0.506) |
| Grade>=3 | 0.006 (0.003-0.013) | 0.072 (0.055-0.093) | 0.137 (0.116-0.160) | 0.148 (0.121-0.180) |
| Increased uric acid | 0.331 (0.284-0.381) | - | 0.461 (0.412-0.512) | - |
| Grade>=3 | 0.081 (0.057-0.113) | - | 0.141 (0.110-0.180) | - |
| Proteinuria | 0.049 (0.035-0.069) | 0.452 (0.416-0.489) | 0.137 (0.111-0.168) | 0.177 (0.147-0.211) |
| Grade>=3 | 0.002 (0.000-0.009) | 0.112 (0.090-0.137) | 0.040 (0.027-0.060) | 0.042 (0.028-0.062) |
| Pyrexia | 0.386 (0.349-0.423) | 0.451 (0.399-0.504) | 0.128 (0.108-0.151) | 0.087 (0.066-0.113) |
| Grade>=3 | 0.009 (0.004-0.020) | 0.024 (0.012-0.046) | 0.011 (0.006-0.020) | 0.004 (0.001-0.013) |
| Hypophosphatemia | 0.239 (0.198-0.286) | - | 0.428 (0.396-0.460) | 0.348 (0.310-0.389) |
| Grade>=3 | 0.061 (0.041-0.091) | - | 0.081 (0.065-0.101) | 0.043 (0.029-0.064) |
| Hand-foot syndrome | 0.031 (0.017-0.054) | - | 0.416 (0.385-0.448) | 0.294 (0.258-0.333) |
| Grade>=3 | 0.011 (0.004-0.028) | - | 0.106 (0.088-0.128) | 0.058 (0.041-0.080) |
| Hypoalbuminemia | - | - | 0.411 (0.370-0.452) | 0.323 (0.285-0.363) |
| Grade>=3 | - | - | 0.016 (0.009-0.031) | 0.007 (0.003-0.018) |
| Dysgeusia | 0.150 (0.117-0.191) | - | 0.402 (0.371-0.434) | 0.258 (0.223-0.296) |
| Grade>=3 | 0.000 (0.000-0.011) | - | 0.004 (0.002-0.011) | 0.002 (0.000-0.010) |
| Increased alkaline phosphatase | 0.369 (0.321-0.420) | - | 0.329 (0.300-0.360) | 0.278 (0.242-0.317) |
| Grade>=3 | 0.019 (0.009-0.040) | - | 0.014 (0.008-0.024) | 0.031 (0.019-0.049) |
| Bilirubin elevation | 0.019 (0.009-0.040) | - | 0.237 (0.211-0.266) | 0.359 (0.320-0.400) |
| Grade>=3 | 0.000 (0.000-0.011) | - | 0.018 (0.012-0.029) | 0.032 (0.021-0.051) |
| Increased amylase | 0.319 (0.273-0.369) | - | 0.349 (0.303-0.399) | - |
| Grade>=3 | 0.039 (0.023-0.064) | - | 0.061 (0.041-0.090) | - |
| Hyponatremia | - | - | 0.316 (0.278-0.356) | 0.347 (0.308-0.387) |
| Grade>=3 | - | - | 0.068 (0.049-0.092) | 0.074 (0.055-0.099) |
| Hemorrhage | 0.092 (0.064-0.130) | 0.332 (0.284-0.384) | - | - |
| Grade>=3 | 0.003 (0.000-0.018) | 0.033 (0.018-0.057) | - | - |
| Hair color changes | 0.011 (0.004-0.028) | - | 0.139 (0.118-0.162) | 0.303 (0.266-0.343) |
| Grade>=3 | 0.000 (0.000-0.011) | - | 0.001 (0.000-0.006) | 0.000 (0.000-0.007) |
| Chills | 0.289 (0.244-0.338) | - | 0.069 (0.048-0.100) | - |
| Grade>=3 | 0.000 (0.000-0.011) | - | 0.011 (0.004-0.027) | - |
| Stomatitus or mucositis | 0.039 (0.023-0.064) | - | 0.285 (0.257-0.315) | 0.139 (0.113-0.170) |
| Grade>=3 | 0.011 (0.004-0.028) | - | 0.017 (0.011-0.028) | 0.007 (0.003-0.018) |
| Vomiting | 0.119 (0.090-0.157) | - | 0.284 (0.256-0.314) | 0.280 (0.244-0.319) |
| Grade>=3 | 0.011 (0.004-0.028) | - | 0.034 (0.024-0.047) | 0.020 (0.011-0.035) |
| Dyspepsia | 0.050 (0.032-0.078) | - | 0.270 (0.242-0.299) | 0.141 (0.114-0.172) |
| Grade>=3 | 0.011 (0.004-0.028) | - | 0.012 (0.007-0.021) | 0.000 (0.000-0.007) |
| Hyperkalemia | - | - | 0.241 (0.207-0.278) | 0.262 (0.227-0.300) |
| Grade>=3 | - | - | 0.018 (0.010-0.033) | 0.031 (0.019-0.049) |
| Abdominal pain | 0.031 (0.017-0.054) | - | 0.193 (0.169-0.220) | 0.262 (0.227-0.300) |
| Grade>=3 | 0.000 (0.000-0.011) | - | 0.018 (0.012-0.029) | 0.022 (0.012-0.037) |
| Influenza-like illness | 0.253 (0.208-0.305) | 0.243 (0.201-0.292) | - | - |
| Grade>=3 | 0.020 (0.009-0.042) | 0.030 (0.016-0.054) | - | - |
| Hypocalcemia | - | - | 0.210 (0.178-0.246) | 0.245 (0.211-0.283) |
| Grade>=3 | - | - | 0.005 (0.002-0.016) | 0.007 (0.003-0.018) |
| Hypomagnesemia | - | - | 0.234 (0.200-0.271) | 0.226 (0.193-0.262) |
| Grade>=3 | - | - | 0.013 (0.006-0.026) | 0.002 (0.000-0.010) |
| Headache | 0.161 (0.135-0.191) | 0.234 (0.192-0.282) | 0.186 (0.163-0.213) | 0.227 (0.194-0.264) |
| Grade>=3 | 0.006 (0.002-0.015) | 0.021 (0.010-0.042) | 0.011 (0.006-0.020) | 0.027 (0.016-0.044) |
| Rash | 0.081 (0.057-0.113) | - | 0.233 (0.207-0.261) | 0.175 (0.146-0.209) |
| Grade>=3 | 0.011 (0.004-0.028) | - | 0.013 (0.007-0.023) | 0.007 (0.003-0.018) |
| Dry skin | 0.061 (0.041-0.091) | - | 0.211 (0.172-0.255) | - |
| Grade>=3 | 0.000 (0.000-0.011) | - | 0.011 (0.004-0.027) | - |
| Thyroid dysfunction | 0.010 (0.005-0.020) | 0.006 (0.002-0.020) | 0.202 (0.177-0.229) | 0.121 (0.096-0.151) |
| Grade>=3 | 0.006 (0.002-0.014) | 0.006 (0.002-0.020) | 0.011 (0.006-0.020) | 0.000 (0.000-0.007) |
| Skin discoloration | 0.011 (0.004-0.028) | - | 0.199 (0.175-0.226) | 0.007 (0.003-0.018) |
| Grade>=3 | 0.000 (0.000-0.011) | - | 0.004 (0.002-0.011) | 0.000 (0.000-0.007) |
| Constipation | 0.039 (0.023-0.064) | - | 0.190 (0.166-0.216) | 0.170 (0.141-0.203) |
| Grade>=3 | 0.000 (0.000-0.011) | - | 0.010 (0.005-0.018) | 0.007 (0.003-0.018) |
| Cough | - | - | 0.181 (0.151-0.215) | 0.153 (0.126-0.186) |
| Grade>=3 | - | - | 0.002 (0.000-0.010) | 0.000 (0.000-0.007) |
| Epistaxis | 0.019 (0.009-0.040) | - | 0.179 (0.155-0.205) | 0.087 (0.066-0.113) |
| Grade>=3 | 0.000 (0.000-0.011) | - | 0.011 (0.006-0.020) | 0.002 (0.000-0.010) |
| Hypermagnesemia | - | - | 0.177 (0.147-0.211) | 0.112 (0.088-0.141) |
| Grade>=3 | - | - | 0.046 (0.031-0.066) | 0.023 (0.014-0.040) |
| Pain in extremity | 0.031 (0.017-0.054) | - | 0.172 (0.149-0.198) | 0.121 (0.096-0.151) |
| Grade>=3 | 0.000 (0.000-0.011) | - | 0.011 (0.006-0.020) | 0.004 (0.001-0.013) |
| Myalgia | 0.169 (0.134-0.212) | - | 0.080 (0.057-0.112) | - |
| Grade>=3 | 0.011 (0.004-0.028) | - | 0.011 (0.004-0.027) | - |
| Back pain | - | - | 0.162 (0.134-0.196) | 0.159 (0.131-0.192) |
| Grade>=3 | - | - | 0.018 (0.010-0.033) | 0.018 (0.010-0.033) |
| Weight loss | 0.130 (0.107-0.157) | 0.157 (0.124-0.199) | 0.085 (0.068-0.104) | 0.152 (0.124-0.184) |
| Grade>=3 | 0.013 (0.007-0.024) | 0.041 (0.025-0.067) | 0.005 (0.002-0.013) | 0.009 (0.004-0.021) |
| Peripheral edema | 0.011 (0.004-0.028) | - | 0.152 (0.130-0.176) | 0.106 (0.083-0.135) |
| Grade>=3 | 0.000 (0.000-0.011) | - | 0.007 (0.003-0.014) | 0.002 (0.000-0.010) |
| Hypokalemia | - | - | 0.150 (0.122-0.182) | 0.141 (0.114-0.172) |
| Grade>=3 | - | - | 0.013 (0.006-0.026) | 0.020 (0.011-0.035) |
| Hypoglycemia | - | - | 0.104 (0.081-0.132) | 0.150 (0.123-0.182) |
| Grade>=3 | - | - | 0.005 (0.002-0.016) | 0.004 (0.001-0.013) |
| Dizziness | - | - | 0.144 (0.117-0.176) | 0.126 (0.101-0.157) |
| Grade>=3 | - | - | 0.005 (0.002-0.016) | 0.009 (0.004-0.021) |
| Dyspnea | 0.098 (0.081-0.118) | 0.139 (0.115-0.166) | 0.143 (0.122-0.167) | 0.137 (0.111-0.168) |
| Grade>=3 | 0.026 (0.018-0.037) | 0.036 (0.024-0.052) | 0.023 (0.015-0.035) | 0.025 (0.015-0.042) |
| Arthralgia | 0.139 (0.107-0.178) | - | 0.114 (0.095-0.136) | 0.139 (0.113-0.170) |
| Grade>=3 | 0.011 (0.004-0.028) | - | 0.010 (0.005-0.018) | 0.016 (0.009-0.031) |
| Alopecia | 0.089 (0.064-0.123) | - | 0.098 (0.080-0.118) | 0.135 (0.109-0.166) |
| Grade>=3 | 0.000 (0.000-0.011) | - | 0.000 (0.000-0.004) | 0.000 (0.000-0.007) |
| Decline in ejection fraction | 0.031 (0.017-0.054) | - | 0.131 (0.100-0.169) | - |
| Grade>=3 | 0.011 (0.004-0.028) | - | 0.029 (0.016-0.052) | - |
| Depression | 0.102 (0.073-0.141) | 0.122 (0.091-0.161) | - | - |
| Grade>=3 | 0.013 (0.005-0.033) | 0.030 (0.016-0.054) | - | - |
| Dry mouth | 0.061 (0.041-0.091) | - | 0.120 (0.091-0.157) | - |
| Grade>=3 | 0.011 (0.004-0.028) | - | 0.000 (0.000-0.010) | - |
| Oral pain | 0.011 (0.004-0.028) | - | 0.112 (0.093-0.134) | 0.070 (0.052-0.095) |
| Grade>=3 | 0.000 (0.000-0.011) | - | 0.004 (0.002-0.011) | 0.002 (0.000-0.010) |
| Insomnia | - | - | 0.109 (0.086-0.138) | 0.105 (0.082-0.133) |
| Grade>=3 | - | - | 0.000 (-0.000-0.007) | 0.000 (0.000-0.007) |
| Flatulence | 0.019 (0.009-0.040) | - | 0.109 (0.082-0.145) | - |
| Grade>=3 | 0.000 (0.000-0.011) | - | 0.000 (0.000-0.010) | - |
| Increased blood LDH | - | - | 0.106 (0.083-0.134) | 0.070 (0.052-0.095) |
| Grade>=3 | - | - | 0.005 (0.002-0.016) | 0.004 (0.001-0.013) |
| Glossodynia | 0.011 (0.004-0.028) | - | 0.101 (0.075-0.136) | - |
| Grade>=3 | 0.000 (0.000-0.011) | - | 0.000 (0.000-0.010) | - |
| Gastroesophageal reflux | 0.011 (0.004-0.028) | - | 0.101 (0.075-0.136) | - |
| Grade>=3 | 0.000 (0.000-0.011) | - | 0.011 (0.004-0.027) | - |
| Erythema | 0.011 (0.004-0.028) | - | 0.101 (0.075-0.136) | - |
| Grade>=3 | 0.000 (0.000-0.011) | - | 0.011 (0.004-0.027) | - |
| Hemorrhage (gastrointestinal) | 0.009 (0.003-0.025) | 0.050 (0.032-0.077) | - | - |
| Grade>=3 | 0.003 (0.000-0.016) | 0.011 (0.004-0.028) | - | - |
| Cardiac ischemia/infarction | 0.000 (0.000-0.011) | 0.014 (0.006-0.032) | 0.040 (0.027-0.060) | 0.020 (0.011-0.035) |
| Grade>=3 | 0.000 (0.000-0.011) | 0.014 (0.006-0.032) | - | - |
| Thrombosis | 0.014 (0.007-0.026) | 0.034 (0.023-0.051) | - | - |
| Grade>=3 | 0.008 (0.003-0.018) | 0.017 (0.010-0.030) | - | - |
| Wound healing complication | 0.010 (0.003-0.029) | 0.015 (0.006-0.034) | - | - |
| Grade>=3 | 0.000 (0.000-0.012) | 0.006 (0.002-0.021) | - | - |
| Cerebrovascular ischemia | 0.003 (0.000-0.016) | 0.014 (0.006-0.032) | - | - |
| Grade>=3 | 0.003 (0.000-0.016) | 0.014 (0.006-0.032) | - | - |
| Pneumonitis/pulmonary infiltrates | 0.012 (0.004-0.029) | 0.008 (0.003-0.024) | - | - |
| Grade>=3 | 0.009 (0.003-0.025) | 0.003 (0.000-0.015) | - | - |
| Perforation (gastrointestinal) | 0.000 (0.000-0.006) | 0.009 (0.004-0.019) | - | - |
| Grade>=3 | 0.000 (0.000-0.006) | 0.007 (0.003-0.017) | - | - |
| Hemorrhage (genitourinary) | 0.003 (0.000-0.016) | 0.008 (0.003-0.024) | - | - |
| Grade>=3 | 0.000 (0.000-0.011) | 0.000 (0.000-0.011) | - | - |
| Left ventricular dysfunction | 0.000 (0.000-0.011) | 0.006 (0.002-0.020) | - | - |
| Grade>=3 | 0.000 (0.000-0.011) | 0.006 (0.002-0.020) | - | - |
| Congestive heart failure | 0.003 (0.000-0.018) | 0.003 (0.000-0.017) | - | - |
| Grade>=3 | 0.000 (0.000-0.012) | 0.003 (0.000-0.017) | - | - |
| Any grade 3, 4, or 5 | 0.544 (0.505-0.582) | 0.705 (0.670-0.738) | 0.734 (0.695-0.769) | 0.744 (0.706-0.778) |
| AE leading to discontinuation of drug | 0.185 (0.158-0.217) | 0.282 (0.237-0.332) | 0.197 (0.173-0.224) | 0.244 (0.210-0.281) |
| AE leading to death | 0.013 (0.008-0.022) | 0.016 (0.009-0.028) | 0.022 (0.014-0.033) | 0.023 (0.014-0.040) |
| SAE | 0.164 (0.127-0.210) | 0.291 (0.245-0.341) | - | - |
